# Family functioning and resilience in children in mainland China: life satisfaction as a mediator

**DOI:** 10.3389/fpsyg.2023.1175934

**Published:** 2023-05-11

**Authors:** Diya Dou, Daniel T. L. Shek, Lindan Tan, Li Zhao

**Affiliations:** ^1^Department of Applied Social Sciences, The Hong Kong Polytechnic University, Kowloon, Hong Kong SAR, China; ^2^Department of Health Policy and Management, West China School of Public Health and West China Fourth Hospital, Sichuan University, Chengdu, China

**Keywords:** family functioning, resilience, life satisfaction, Chinese children, positive youth development, mediator

## Abstract

**Introduction:**

Grounded in the perspective of “Positive Youth Development” (PYD), resilience is an important developmental asset shaping human development. Although many studies have examined the impact of resilience on child developmental outcomes, relatively few studies have focused on the predictors of resilience, in particular familial antecedents of resilience in Chinese children and adolescents. In addition, the degree to which life satisfaction contributes to the mechanism by which family functioning impacts the development of children’s resilience over time needs to be clarified. Besides, there is a scarcity of studies that incorporate family functioning, resilience as well as life satisfaction in a single comprehensive investigation to analyze the mediating impact of life satisfaction on the linkage between family functioning and resilience under COVID-19.

**Methods:**

The study investigated the predictive role of family functioning on resilience as well as the mediating effect of life satisfaction within the context of COVID-19, using data gathered in two waves before the onset of the pandemic and after the resumption of school during the pandemic, with 6 months apart. We employed the 33-item “Chinese Family Assessment Instrument” to evaluate family functioning, the 7-item “Chinese Resilience Scale” to assess resilience, and the “Satisfaction with Life Scale” with 5 items to measure life satisfaction.

**Results:**

As per the responses of 4,783 students in Grades 4 through 7 recruited in Sichuan, China, family functioning significantly predicted resilience concurrently and longitudinally. After controlling for resilience scores in Wave 1, results demonstrated that family functioning examined in Wave 1 predicted an increase in resilience reported in Wave 2. In addition, family functioning significantly predicted life satisfaction, which also significantly predicted resilience. Multiple regression using PROCESS analyses indicated that life satisfaction mediated the predictive relationship between family functioning and child resilience.

**Discussion:**

The findings spotlight the significant involvement of family functioning as well as life satisfaction in shaping children’s resilience in the Chinese context. The study also supports the hypothesis that perceived satisfaction with life serves as a mediator between family functioning and child resilience, suggesting interventions and support should concentrate on the family level for enhancing resilience in children.

## 1. Introduction

Walsh (2002) defined resilience as “the ability to withstand and rebound from adversity” (p. 130). According to Shek et al. (2017), resilience is the ability to positively adjust to stressful life events and maintain healthy reactions in stressful contexts. It is a multi-dimensional concept including three fundamental elements: exposure to stressful events, positive adjustment to adversity, and sustainability. Resilience is an important internal developmental asset that helps adolescents face various developmental challenges, such as identity formation, stress management, and relationship formation. Strong resilience in children and adolescents is associated with better self-autonomy, critical thinking skills, social competence, a sense of purpose, and problem-solving abilities (Zolkoski and Bullock, 2012) as well as better development in various life domains (Wu et al., 2017). Under COVID-19, resilience can help children and adolescents overcome various adaptation issues, such as anxiety, social isolation, economic stress, and uncertainty. Rania et al. (2022a) argued that individual empowerment, which involves building individual resilience, contributes to group resilience in response to the collective trauma resulting from the pandemic. Therefore, cultivating resilience is a promising strategy for helping children and adolescents adapt positively to internal and external changes and challenges.

According to the Developmental Systems Theory (DST) that advocates the influence of different systems on developmental outcomes (Ford and Lerner, 1992), cultivating resilience involves three main factors, including personality traits, family system characteristics, and the broader social context such as schools and communities (Zolkoski and Bullock, 2012). Given the collectivistic nature of Chinese culture (Wu and Tseng, 1985; Wu et al., 2017), family system characteristics appear to play a more prominent role (Wu et al., 2017), as highlighted by “the persistence of a closely knit family as the center of Chinese culture” (Wu and Tseng, 1985, p. 8). Furthermore, as “resilience is not a simple linear causal process in which limited to the strength that leads directly to a good developmental outcome” (Akbar et al., 2014, p. 517), there is a need to explore the interactions between the diverse protective factors and multiple processes involved in resilience. In this study, we examined the predictive effect of family functioning on children’s resilience under the context of COVID-19, with life satisfaction proposed as a mediating factor.

### 1.1. Family functioning and resilience

Scholars have conceptualized different dimensions of family functioning. Patterson (2002) proposed that family functioning includes economic support, family formation, socialization, and protection of vulnerable members. Epstein et al. (2003) identified six dimensions of family functioning: communication, problem-solving, role functioning, affective responsiveness and involvement, and behavior control. Olson (2011) discussed both the positive aspects, such as cohesion and flexibility, and negative aspects, such as disengagement, enmeshment, rigidity, and chaos, of family functioning. Shek (2002a) developed the “Chinese Family Assessment Instrument” (C-FAI) to assess both systemic attributes, such as harmony, mutuality, and absence of conflict, and dyadic attributes of parent–child relations, including parental concern and parental control, as indicators of family functioning.

The connection between family functioning and personal resilience has theoretical support. According to the perspective of “Positive Youth Development” (PYD) (Shek et al., 2017), family functioning is an environmental asset for child and youth development. Family functioning processes delineated in the Circumplex model are considered to “underpin resilient outcomes among family members” (Oshri et al., 2015, p. 46). In addition, building family resilience, as advocated by the “Family Resilience Theoretical Framework,” seeks to strengthen the family as a dynamic organism capable of fostering individual resilience of all its members (Walsh, 1996), demonstrating the protective impact of family function on individual resilience.

Empirical evidence also supports the association between healthy family functioning and the resilience of children and adolescents. According to a study conducted by Wyman et al. (1992) on 2,069 primary school students, resilient students reported a more stable home environment, more positive relationships with caregivers, and greater consistency in family discipline. Using interview data collected from 77 U.S. adolescents whose parents were HIV-infected, Rosenblum et al. (2005) demonstrated that positive family functioning was positively associated with resilience and negatively associated with substance abuse and peer deviance. Similarly, Sahanowas and Halder (2019) revealed family problem-solving and roles predicted resilience among Indian undergraduate students (*N* = 490).

### 1.2. Family functioning and life satisfaction

As a cognitive constituent of subjective well-being (Andrews and Withey, 1976), life satisfaction is conceptualized as a “conscious cognitive judgment of one’s life in which the criteria for judgment are up to the person” (Pavot and Diener, 1993, p. 164). Generally speaking, the level of a person’s life satisfaction is determined by the degree to which the perceived quality of life meets self-defined criteria.

Existing literature has demonstrated that family factors, such as parental control, family communication, family migration, cohesion, and adaptability, are crucial for adolescents to achieve and enhance life satisfaction. Based on 255 Italian adolescents aged 15–17 years, Cacioppo et al. (2013) found a positive association between family functioning and overall life satisfaction among adolescents. Using a sample of 703 Chinese students who had experienced family immigration from rural to urban areas, Yuan et al. (2019) found that family communication, cohesion, and adaptability were positively related to students’ life satisfaction. Researchers also found that negative aspects of family functioning have destructive effects on adolescent life satisfaction. For example, Cacioppo et al. (2013) reported that teenagers’ overall life satisfaction was negatively correlated with perceived parental psychological control. Rania et al. (2022b) conducted a survey with 560 Italian parents and reported that increased family conflict could significantly undermine family well-being, especially when the fragility of families has been exposed by the COVID-19 pandemic that acts like a “magnifying glass.” In the Chinese context, Shek (2002b, 2005), and Shek and Liu (2014) have conducted a series of investigations and found a positive association between family functioning and adolescent development and well-being. For example, using the “Chinese Family Assessment Instrument,” Shek (2002b) examined juvenile adaptation in 1,519 Hong Kong teenagers and revealed that family functioning was significantly related to adolescent life satisfaction.

### 1.3. Life satisfaction and resilience

Research on resilience has gained prominence in the field of positive psychology in the last decade (Shek et al., 2017). The positive psychology paradigm criticizes that “psychological practice focuses almost exclusively on pathology” (Faller, 2001, p. 7), and emphasizes promoting life satisfaction through building strengths such as resilience and self-efficacy (Gilman and Huebner, 2003). Guided by positive psychology, researchers have examined the relationship between resilience and psychological well-being and found that resilient individuals tended to report higher life satisfaction and lower levels of ill-being, such as psychological anxiety. For instance, a study conducted by Beutel et al. (2010) with 2,144 Germans revealed that resilience was positively correlated with general life satisfaction and self-esteem, and negatively correlated with depression and anxiety. Similarly, Akbar et al. (2014) surveyed 100 Nomads and found a significant positive correlation between life satisfaction and resilience.

Despite numerous studies focusing on the positive effect of resilience on psychological well-being, life satisfaction may also influence resilience. As argued by Proctor et al. (2009), “life satisfaction is more than just an outcome of various psychological states, it is also an influential predictor of psychosocial systems” (p. 604). Based on a sample of 929 adults in Turkey during the COVID-19 pandemic, Karataş and Tagay (2021) found that life satisfaction, meaning in life, and the absence of traumatic experiences positively predicted resilience. Similar findings were reported in adolescent samples. Sahin Baltaci and Karataş (2015) reported that life satisfaction and perceived social backing were significant predictors of resilience among 386 Turkish secondary school students. In addition, Yakici and Zeliha (2018) found that life satisfaction had a positive predictive effect on resilience among 659 Turkish university students.

### 1.4. Family functioning, life satisfaction, and resilience

A few studies have examined the relationships between family functioning, life satisfaction, and resilience in a single study. This issue is important because studies reveal close relationships between these constructs, such as the mediating effect of resilience in linking family functioning and life satisfaction. For example, Azpiazu Izaguirre et al. (2021) surveyed 1,188 middle school students in Spain and found that resilience partially mediated the relationship between family support and satisfaction with life. Similarly, Zarei and Fooladvand (2022) found that family functioning positively affected life satisfaction *via* the mediation of students’ resilience among 480 Iranian female college students under COVID-19.

While research findings highlight the close relationships between family functioning, resilience, and life satisfaction, as well as the mediating effect of resilience and its subsequent developmental outcomes, less is known about the role of life satisfaction as a mediator between family functioning and resilience. Empirical investigations have established that life satisfaction serves a mediation role between family factors and individual well-being. For instance, based on a survey of 1,086 employees in the United States, Rode et al. (2007) revealed that satisfaction with their life mediated the effect of family-work role conflict on exit from the workplace. Besides, Urbanova et al. (2019) surveyed 2,844 Slovak adolescents and found that life satisfaction mediated part of the connection between the family socioeconomic situation and Internet overexposure. Nevertheless, there are few studies exploring the inter-relationships amongst perceived family functioning, life satisfaction, and resilience in adolescents, particularly the mediating role of life satisfaction.

### 1.5. The present study

There are several research gaps in the literature. First, while most related studies are Western studies, few attempts in Chinese societies have examined the predictors of child resilience, particularly family antecedents of resilience in children (Shek et al., 2005; Shek, 2010). In addition, Proctor et al. (2009) reviewed 141 empirical studies on youth life satisfaction and found that most research has been based on American culture, with most assessment measures constructed and validated in the U.S. sample, strongly supporting the need to conduct non-Western and cross-cultural research. Second, few studies have examined how well families are functioning in relation to resilience among adolescents during the course of the COVID-19 pandemic. The pandemic induced infection-related anxiety, social alienation, economic stress and uncertainty, posing many adaptation challenges to the general public (Shek et al., 2022a). Adolescents experiencing the “storm and stress” phase of puberty (Rosenblum et al., 2005, p. 584) may become more vulnerable when confronted with difficulties caused by the pandemic, such as the closure of schools and the unemployment of family members. Third, the role that life satisfaction plays in mediating the family functioning-resilience relationship remains understudied. Fourth, the vast majority of existing studies in this field are cross-sectional in nature, with relatively few longitudinal and large-sample studies. To address these research gaps, the present study aimed to investigate the mediating role of children’s perceived life satisfaction between family functioning and resilience among Chinese children utilizing two waves of data collected separately before and during COVID-19. The research questions and hypotheses are presented below.

Research Question 1: Does family functioning predict resilience in children concurrently and longitudinally? Based on the “Family Resilience Theoretical Framework” (Walsh, 1996) as well as previous studies (Rosenblum et al., 2005; Sahanowas and Halder, 2019; Shek et al., 2022b,c), we proposed two hypotheses:

*Hypothesis 1a*: family functioning would have a concurrent relationship with the child and adolescent resilience.

*Hypothesis 1b*: family functioning would have a longitudinal relationship with the child and adolescent resilience.

Research Question 2: Does family functioning predict satisfaction with life concurrently and longitudinally? With reference to the family functioning models (e.g., McMaster model), and past studies (Cacioppo et al., 2013; Botha and Booysen, 2014; Song et al., 2022), we developed two hypotheses:

*Hypothesis 2a*: family functioning would have a positive concurrent prediction of children’s life satisfaction.

*Hypothesis 2b*: family functioning would positively predict children’s life satisfaction across time.

Research Question 3: Does life satisfaction predict resilience in children concurrently and longitudinally? As suggested in previous studies (Sahin Baltaci and Karataş, 2015; Yakici and Zeliha, 2018; Karataş and Tagay, 2021), we established two hypotheses as follows:

*Hypothesis 3a*: life satisfaction would have a positive concurrent prediction of resilience in children.

*Hypothesis 3b*: life satisfaction would positively predict resilience in children longitudinally.

Research Question 4: Does life satisfaction mediate the predictive impact of family functioning on resilience in children over time? In light of the previous studies that supported life satisfaction as a mediator of family factors and various individual psychological outcomes (Rode et al., 2007; Urbanova et al., 2019), we proposed the following hypothesis:

*Hypothesis 4*: children’s satisfaction towards their life investigated at Wave 2 would mediate the prediction of family function reported in Wave 1 and child resilience examined at Wave 2.

## 2. Methods

### 2.1. Participants and procedures

We collected data from five schools in Chengdu, China through the cluster sampling method. As for location, one school in the city center, two in the southern suburbs, and two in the northern suburbs of Chengdu were selected. Concerning school level, two schools were elementary schools, one was junior high school, and two admitted both elementary and secondary school students, where the survey was open to all students. The initial round of data was obtained prior to the outbreak of the pandemic (i.e., from December 2019 to January 2020). The second round of data was gathered from June to July 2020 after schools resumed. In both waves of the survey, students filled out an identical questionnaire during class. For each class, a trained researcher was present during data collection to explain the study’s purpose and answer students’ questions, if any arose. Prior to participation, schools, parents, and students, all gave their informed consent accordingly. The survey received ethical approval from Sichuan University. The current study mainly focused on students from Grades 4 to 7. In total, 5,681 students participated in Wave 1 and 4,783 in Wave 2. The attrition rate was 15.8%, which is not particularly high and comparable to that of other large-scale surveys conducted with Chinese children and adolescents (e.g., China Family Panel Studies, CFPS). Some students were absent from Wave 2 data collection due to illness or other personal reasons. Attrition analysis revealed no notable differences in demographic characteristics between the matched respondents (i.e., those who participated in both Wave 1 and Wave 2) and the drop-out sample. The final dataset consisted of 4,783 students, with 47.2% girls (*n* = 2,259) and 51.7% boys (*n* = 2,472), with a 12-year average age at Wave 1. Among them, 98.2% of the participants were Han, while 0.7% were minority groups.

### 2.2. Measures

#### 2.2.1. Family functioning

Family functioning was measured by the “Chinese Family Assessment Instrument” (C-FAI) (Shek, 2002a). Previous studies involving Chinese children have established the C-FAI’s good psychometric properties in terms of validity, reliability, and measurement invariance (Shek and Ma, 2010a). The C-FAI consisted of 33 items that measured five aspects of family functioning, including “communication” (9 items), “mutuality” (12 items), “parental concern” (3 items), “parental control” (3 items), and “conflict and harmony” (6 items). Some sample items included “parents often communicate with children” (communication), “family members support each other” (mutuality), and “parents care about their children” (parental concern). We employed a 5-point Likert scale in all the items, where 1 indicating the most similar and 5 the most dissimilar. Positive items were reverse-coded so that higher scores indicated better family functioning. The C-FAI in the current investigation demonstrated good reliability, with Cronbach’s alpha of 0.936 and of 0.944 at Wave 1 and Wave 2, respectively (refer to Table 1).

**Table 1:** Descriptive, correlational analyses, and reliability of scales.

Measures	α	Inter-item correlations	Mean	SD	Correlations							
					1	2	3	4	5	6	7	8
Age	--	--	12	1.16	--							
Gender^a^	--	--	--	--	−0.011	--						
Ethnic group^b^	--	--	--	--	0.004	−0.016	--					
RE W1	0.827	0.448	5.38	0.73	−0.045**	0.021	−0.003	--				
LS W1	0.759	0.429	4.5	1.11	−0.066***	−0.005	0.001	0.449***	--			
FFT W1	0.936	0.324	4.13	0.71	−0.009	0.058***	0.003	0.410***	0.345***	--		
RE W2	0.882	0.558	5.3	0.88	−0.111***	−0.013	−0.006	0.416***	0.344***	0.287***	--	
LS W2	0.814	0.500	4.41	1.22	−0.105***	−0.063***	0.015	0.299***	0.444***	0.268***	0.505***	--
FFT W2	0.944	0.373	4.12	0.74	−0.082***	0.040**	−0.004	0.303***	0.275***	0.511***	0.380***	0.388***

^a^1 = male, 2 = female; ^b^1 = The Hans, 2 = Minorities; W1, Wave 1; W2, Wave 2; RE, resilience; LS, life satisfaction; FFT, family function. **p < 0.01; ***p < 0.001.

#### 2.2.2. Life satisfaction

Life satisfaction was evaluated by the “Satisfaction with Life Scale” (SWLS) (Diener et al., 1985). Five statements reflecting a person’s global life satisfaction were presented to the students to indicate the range of their agreement, such as “In most ways my life is close to my ideal,” and “The conditions of my life are excellent.” Responses were rated on a “6-point Likert scale” ranging from 1 for “strongly disagree” to 6 for “strongly agree.” Higher scores signify elevated levels of satisfaction with life. The instrument has previously exhibited sound reliability and validity when administered to Chinese adolescents in past research (Zhu and Shek, 2020). For Waves 1 and 2 of the current study, Cronbach’s alpha values were 0.759 and 0.814, respectively.

#### 2.2.3. Resilience

Students’ resilience was scaled by the “Resilience Subscale” of the “Chinese Positive Youth Development Scale” (CPYDS) developed and validated by Shek and Ma (2010b). This scale comprises 15 sub-scales evaluating key PYD attributes outlined by Catalano et al. (2004), such as “resilience,” “emotional competence,” “cognitive competence,” and “self-efficacy.” The resilience sub-scale consisted of six items that evaluated students’ capacities to adapt and rebound from adverse experiences and arduous challenges on a six-point Likert scale (1 = “strongly disagree,” 6 = “strongly agree”), with higher scores reflecting greater resilience. A case in point for one item is “I do not give up easily when facing difficulties.” The Cronbach’s alpha was 0.827 at Wave 1 and 0.882 at Wave 2 in the present investigation.

#### 2.2.4. Covariates

Gender, age, and ethnicity were all considered covariates in this study and were controlled in all models and analyses. Previous studies have indicated that these variables are likely to be correlated with family functioning and adolescent development (Shek et al., 2022b,c). As Sichuan Province’s diverse population includes a number of minority groups, considering ethnicity helps in examining potential differences in experiences and perceptions between students from different ethnic groups. We created dichotomous variables for ethnic group (1 = Hans, 2 = Minority) and gender (1 = boy, 2 = girl).

### 2.3. Analysis

The analyses were conducted using SPSS 28.0. Initial data examination involved descriptive statistical analyses to assess the standard deviations, means, and correlations of the variables. Subsequently, hierarchical multiple regression analyses were executed to investigate the contemporaneous and longitudinal associations between the variables of interest, as described in Research Questions 1, 2, and 3. Additionally, we controlled for resilience at Wave 1 when exploring how resilience is affected longitudinal by family functioning as well as satisfaction with life. The mediation effect of life satisfaction (Research Question 4) was also analyzed using the PROCESS macro (Hayes, 2018) in SPSS. We calculated bias-corrected 95% confidence intervals using 5,000 re-samplings.

## 3. Results

### 3.1. Descriptive results

The descriptive statistics, reliability, and correlations of variables measured at two time points are exhibited in Table 1. With Cronbach’s alphas greater than 0.759, all scales showed sound reliability. Separate analyses of the reliability at each grade also showed that the reliability measures were good at each grade, suggesting that the responses were not random (refer to Table 2). As expected, resilience was positively correlated with satisfaction with life and family functioning concurrently and longitudinally, with *r*s ranging between 0.299 and 0.505, *p*s < 0.001 (see Table 1). Age was negatively correlated with resilience with a small effect size (*r* = −0.045 at Wave 1, *r* = −0.111 at Wave 2, *p*s < 0.001), denoting that younger students reported higher levels of resilience. Although gender was not significantly related to resilience, girls tended to report better family functioning at both Waves (*r* = 0.058 at Wave 1, *r* = 0.040 at Wave 2, *p*s < 0.001) and lower life satisfaction compared to boys at Wave 2 (*r* = −0.063, *p* < 0.001), with small effect size. Ethnicity did not exhibit a statistically significant correlation with the research variables.

**Table 2:** Reliability of scales by grade.

Measures	Grade 4		Grade 5		Grade 6		Grade 7	
	Omega	*α*	Omega	*α*	Omega	*α*	Omega	*α*
FFT W1	0.80	0.78	0.82	0.80	0.84	0.83	0.82	0.80
FFT W2	0.79	0.77	0.83	0.81	0.82	0.81	0.85	0.83
LS W1	0.72	0.67	0.76	0.72	0.80	0.77	0.86	0.85
LS W2	0.78	0.75	0.81	0.79	0.86	0.84	0.87	0.86
RE W1	0.72	0.71	0.79	0.78	0.86	0.86	0.90	0.89
RE W2	0.78	0.78	0.85	0.85	0.90	0.90	0.93	0.93

RE, resilience; LS, life satisfaction; FFT, family functioning; W1, Wave 1; W2, Wave 2.

### 3.2. The predictive effect of family functioning and life satisfaction on resilience

After controlling for age, gender, and ethnicity, we found significant concurrent positive effects of family functioning on resilience at Wave 1 (*β =* 0.41, *p* < 0.001, Cohen’s *f*^2^ = 0.166, see Table 3) and Wave 2 (*β =* 0.38, *p* < 0.001, Cohen’s *f*^2^ = 0.163, see Table 3). Similar results were observed with respect to the prediction of satisfaction with life on resilience (Wave 1: *β =* 0.45, *p* < 0.001, Cohen’s *f*^2^ = 0.249; Wave 2: *β =* 0.50, *p* < 0.001, Cohen’s *f*^2^ = 0.327, see Table 3). Both family functioning and life satisfaction at Wave 1 exhibited statistically significant longitudinal influence on resilience at Wave 2 (*β* was 0.29 and 0.34, *p*s < 0.001, Cohen’s *f*^2^ was 0.091 and 0.129 for family functioning and life satisfaction, respectively, see Table 4). Furthermore, when Wave 1 resilience was added into the model, family functioning and satisfaction with life at Wave 1 continued to have significant positive predictions on resilience at Wave 2 over time (*β* was 0.15 and 0.19, *p*s < 0.001, Cohen’s *f*^2^ was 0.018 and 0.031 for family functioning and life satisfaction, respectively, refer to Table 4). The findings provided support for Hypotheses 1a, 1b, 3a, and 3b.

**Table 3:** Cross-sectional regression analyses for RE.

Model	Predictors	RE (Wave 1)					RE (Wave 2)				
		*β*	*t*	Cohen’s *f^2^*	*R*^2^ change	*F* change	*β*	*t*	Cohen’s *f^2^*	*R*^2^ change	*F* change
1	Age	−0.04	−3.04**	0.002	0.002	3.75*	−0.11	−7.66***	0.013	0.013	19.89***
	Gender^a^	0.02	1.38	0.000			−0.01	−0.95	0.000		
	Ethnic group^b^	0.00	−0.14	0.000			−0.01	−0.46	0.000		
2	FFT	0.41	30.58***	0.199	0.166	935.32***	0.38	27.83***	0.163	0.140	774.24***
	LS	0.45	34.21***	0.249	0.199	1170.21***	0.50	39.42***	0.327	0.247	1554.05***

In model 2, control variables were statistically controlled; measures of family function at Wave 1 and Wave 2 were included as predictors to predict resilience at Wave 1 and Wave 2, respectively. ^a^1 = male, 2 = female; ^b^1 = The Hans, 2 = Minorities; RE, resilience; FFT, family function; LS, life satisfaction. *p < 0.05; **p < 0.01; ***p < 0.001.

**Table 4:** Longitudinal regression analyses for RE.

Model	Predictors	RE (W2)					RE (W2)				
		*β*	*t*	Cohen’s *f^2^*	*R*^2^ change	*F* change	*β*	*t*	Cohen’s *f^2^*	*R*^2^ change	*F* change
1	Age	−0.111	−7.59***	0.012	0.013	19.59***	−0.091	−6.87***	0.008	0.170	960.70***
	Gender^a^	−0.015	−1.03	0.000			−0.025	−1.88	0.001		
	Ethnic group^b^	−0.007	−0.47	0.000			−0.007	−0.50	0.000		
	W1 RE						0.412	31***	0.204		
2	W1 FFT	0.29	20.68***	0.091	0.083	427.67***	0.15	10.08***	0.018	0.018	101.68***
	W1 LS	0.34	24.63***	0.129	0.114	606.44***	0.19	13.24***	0.031	0.030	175.38***

In model 2, control variables were statistically controlled. ^a^1 = male, 2 = female; ^b^1 = The Hans, 2 = Minorities; RE, resilience; FFT, family function; LS, life satisfaction. ***p < 0.001.

### 3.3. The predictive effect of family functioning on life satisfaction

Results of hierarchical multiple regression analyses indicated that after controlling for covariates, family functioning significantly and positively affected life satisfaction concurrently (Wave 1: *β* = 0.35, *p* < 0.001, Cohen’s *f*^2^ = 0.135; Wave 2: *β* = 0.39, *p* < 0.001, Cohen’s *f*^2^ = 0.173, see Table 5). Additionally, Wave 1 family functioning positively predicted life satisfaction at Wave 2 (*β* = 0.27, *p* < 0.001, Cohen’s *f*^2^ = 0.081, see Table 6). This prediction remained significant even after controlling for life satisfaction at Wave 1 (*β =* 0.14, *p* < 0.001, Cohen’s *f*^2^ = 0.017, see Table 6). There was evidence supporting Hypotheses 2a and 2b.

**Table 5:** Cross-sectional regression analyses for LS.

		LS (Wave 1)					LS (Wave 2)				
Model	Predictors	*β*	*t*	Cohen’s *f^2^*	*R*^2^ change	*F* change	*β*	*t*	Cohen’s *f^2^*	*R*^2^ change	*F* change
1	Age	−0.07	−4.54***	0.004	0.004	6.92***	−0.11	−7.3***	0.011	0.015	24.39***
	Gender^a^	−0.01	−0.41	0.000			−0.06	−4.41***	0.004		
	Ethnic group^b^	0.00	0.11	0.000			0.01	0.95	0.000		
2	FFT	0.35	25.21***	0.135	0.119	635.34***	0.39	28.67***	0.173	0.147	821.84***

In model 2, control variables were statistically controlled; measures of FFT at Wave 1 and Wave 2 were included as predictors to predict LS at Wave 1 and Wave 2, respectively. ^a^1 = male, 2 = female; ^b^1 = The Hans, 2 = Minorities; FFT, family function; LS, life satisfaction. ***p < 0.001.

**Table 6:** Longitudinal regression analyses for LS.

Model	Predictors	LS (W2)					LS (W2)				
		*β*	*t*	Cohen’s *f^2^*	*R*^2^ change	*F* change	*β*	*t*	Cohen’s *f^2^*	*R*^2^ change	*F* change
1	Age	−0.106	−7.28***	0.011	0.015	24.31***	−0.076	−5.8***	0.006	0.193	1128.48***
	Gender^a^	−0.064	−4.4***	0.004			−0.062	−4.77***	0.004		
	Family intactness^b^	0.014	0.95	0.000			0.010	0.80	0.000		
	**W1 LS**						0.440	33.59***	0.239		
2	W1 FFT	0.27	19.48***	0.081	0.075	379.54***	0.14	10.03***	0.017	0.017	100.52***

In model 2, control variables were statistically controlled. ^a^1 = male, 2 = female; ^b^1 = The Hans, 2 = Minorities; FFT, family function; LS, life satisfaction. ***p < 0.001.

### 3.4. The mediation effects of life satisfaction

The mediation analysis *via* PROCESS findings is summarized in Table 7. Results showed that life satisfaction mediated the prediction of family functioning examined in Wave 1 on resilience in Wave 2. The indirect effect of family functioning on resilience through satisfaction with life was significant (*β =* 0.12, *p* < 0.001, see Table 7). The total effect of family functioning on resilience was also examined as significant (*β* = 0.29, *p* < 0.001), with the inclusion of the mediator the effect was still rendered a significant effect (*β* = 0.17, *p* < 0.001). These findings indicate that the association between children’s perceived family functioning and individual resilience was partially mediated by satisfaction with life, supporting Hypothesis 4.

**Table 7:** Longitudinal mediating effect analyses of LS at Wave 2 (the mediators) for the effect of FFT at Wave 1 on RE at Wave 2.

Regression models summary			
	*β*	*SE*	*t*
Total effect of IV (FFT) on DV (RE)	0.29	0.02	20.68^***^
IV (FFT) to Mediator (LS)	0.27	0.02	19.48^***^
Mediator (LS) to DV (RE)	0.45	0.01	34.80^***^
Direct effect of IV (FFT) on DV (RE)	0.17	0.02	12.75^***^
Mediating effect	Point estimate	Bootstrapping (BC 95% CI)	
		Lower	Upper
	0.12^***^	0.11	0.14

RE, resilience; FFT, family function; LS, life satisfaction. ***p < 0.001.

## 4. Discussion

The present study has several unique features. First, how Chinese children’s resilience is shaped by factors at different levels has not been fully investigated, particularly the longitudinal influence of the family system on child development (Shek et al., 2019, 2020). Hence, this research advances comprehension of the intricate interplay between family dynamics and the development and flourishing of children by adopting a multi-dimensional measure of family functioning and using a longitudinal design. Second, this study focused on children with certain demographic characteristics in China’s Sichuan province using a relatively large sample size. This contributes to our understanding of the related issues, and thus informs policies and interventions tailored to their specific developmental needs. Third, this study incorporated a mediational model, which allows for the examination of the underlying mechanisms through which family functioning influences children’s development. Finally, this study was performed in the COVID-19 setting which is different from the non-COVID-19 context. As COVID-19 has exerted much stress on children and adolescents and their families (Shek, 2021a; Shek et al., 2023), this investigation constitutes an interesting addition to the discussion on this issue in the pandemic context.

For Research Question 1, the finding supports that children growing up in supportive, harmonious, and caring families are more inclined to stay resilient when exposed to adversity. The finding is congruent with prior studies demonstrating a beneficial link between healthy family functioning and children’s resilience (e.g., Rosenblum et al., 2005; Wong, 2008; Sahin Baltaci and Karataş, 2015). For example, Wong (2008) reported that adolescent resilience was predicted by elevated perceptions of autonomous support and parental involvement. A survey conducted by Wu et al. (2014) with Chinese migrant youth also showed that family support predicted children’s resilience, which in turn influenced their educational outcomes, such as academic effort and drop-out intention. As suggested by Self-Determination Theory (SDT), a supportive and harmonious family environment can help children develop a sense of personal control, build positive interpersonal connections, and strong confidence, which are all essential protective factors for resilience (Joussemet et al., 2008). It is noteworthy that while there are studies examining the influence of dyadic parent–child relational qualities on child resilience, relatively little research as to the effects of systemic family functioning on children’s resilience has been conducted.

As to Research Question 2, the finding supports the concurrent and longitudinal prediction of family functioning on life satisfaction among children, which is in alignment with earlier studies (e.g., Cacioppo et al., 2013; Yuan et al., 2019). Chang et al. (2003) reported that perceived parental warmth and autonomy positively influenced the life satisfaction of their children, covering both kids and youth, in the Chinese context. Based on the Ecological System Theory (Bronfenbrenner, 1992), family settings are considered the most proximate and influential external circumstance on a child’s growth and flourishing throughout childhood and early adolescence. Children raised in warm and caring family contexts receive emotional assistance to manage problems, resulting in reduced risks for unfavorable developmental outcomes and elevated rates of global life satisfaction.

Regarding Research Question 3, the study demonstrates that life satisfaction also predicted resilience in children concurrently and longitudinally, which is in alignment with the results of former studies (e.g., Sahin Baltaci and Karataş, 2015). For example, Karataş and Tagay (2021) found that life satisfaction positively and substantially predicted resilience. Henderson et al. (2013) also found that hedonic activity helped regulate emotion and would predict positive affect and vitality. Children who are contented with their lives are less prone to dwell on negative emotions, showing lower anxiety and depression symptoms and preserving positive coping resources. As argued by Cattan (2009), children who rated their life satisfaction at higher scores perceive a lower level of stress and possess a more positive outlook on the future and difficult conditions, which benefits their resilience development.

For Research Question 4, the results demonstrated that life satisfaction mediates the link between children’s reported family functioning and children’s resilience, which is consistent with other findings evincing close relationships between family dynamics, children’s well-being and positive growth outcomes (Azpiazu Izaguirre et al., 2021; Zarei and Fooladvand, 2022). This result also aligns with previous research revealing an indirect influence of intimate relationships with parents on resilience mediated by life satisfaction (Khani et al., 2016). According to the Ecological Systems Theory (Bronfenbrenner, 1992), a child’s development is shaped by the interactions of a complex system comprised of interconnected environments at various levels, including the child’s characteristics, immediate family contexts, and broader cultural and social conditions. Children’s life satisfaction *de facto* reflects their perceptions of the interactions between environments and suggests whether these environments can fulfil their developmental needs. Since few investigations have integrated family functioning, satisfaction with life, and resilience in a single study simultaneously, the present study is the pioneer in nature.

The present study presents theoretical implications. It highlights the significance of a well-functioning family in nurturing resilience and extensive well-being in Chinese children. According to prior studies, family functioning can significantly influence children’s well-being, proactive growth, and maladaptive behaviors (Shek, 2002b, 2005; Shek and Liu, 2014). As few studies have focused on the effects of family functioning on children’s resilience, the present study contributes to the development of theory in this field. Additionally, it highlights the importance of global satisfaction with life serving as mediation of the connection between family functioning and youth development. The discovery sheds light on how the functioning of a family impacts the well-being of children. Moreover, it suggests that contentment with life plays a significant role in determining how family functioning affects the development of children. This result is consistent with the Developmental Systems Theory (Ford and Lerner, 1992) and Ecological System Theory (Bronfenbrenner, 1992), which hold that a child’s subjective well-being is a pivotal factor in the connection between perceived environmental circumstances and personal development.

This study has practical implications. It emphasizes the importance of family dynamics on children’s life satisfaction and resilience, hence suggesting the need for family-based interventions that not only build children’s resilience at the individual level but also enhance parent–child relationships at the family level. According to the Ecological System Theory (Bronfenbrenner, 1992), cultural and social norms are also significant surroundings that interact with the functioning of the family and the development of the child. However, traditional Chinese culture emphasizes respect for elders and authority, which may discourage children from expressing their opinions or emotions openly to their parents (Dou et al., 2020). Similarly, parents may conceal their concerns and worries from their children. These communication challenges can result in misunderstandings and conflicts, hampering the ability of families to properly support and encourage their children’s stress coping when faced with adversity. As family-based interventions help to promote effective family communication, concern, and care among family members, and provide resources for families to overcome cultural barriers to effective communication (Shek, 2008), we have to take the unique attributes of Chinese families into account.

In addition, it highlights the essential function of satisfaction with life in fostering resilience in children. Possessing higher levels of satisfaction with life, which serve as a form of psychological well-being, can help children feel more confident and determined to overcome obstacles. Besides, Chinese parents frequently adopt a strict parenting style because discipline is considered key to children’s future success. Employing data gathered from 550 Chinese parents, Leung and Shek (2013) found that paternal expectation of offspring’s future prospects constitutes the strongest predictor of family functioning. Yet, overly strict parenting frequently results in a sole focus on children’s academic accomplishment and a disregard for their subjective well-being, which increases academic stress and other maladaptive behaviors (Suldo and Huebner, 2004). Besides, different stakeholders highlighted that programs promoting positive psychological attributes are inadequate in Hong Kong (Shek et al., 2021). Hence, programs should be designed to help parents realize the critical nature of children’s well-being and its beneficial correlation with academic resilience (Zeng et al., 2022).

Furthermore, since “resilience research has never strayed far from its translational agenda” (Masten, 2011, p. 493), the present findings are also relevant to other non-Chinese cultural contexts, particularly under COVID-19. As COVID-19 has a strong negative impact on mental health (Shek, 2021a,b; Shek et al., 2023), how to promote family functioning and life satisfaction are important keys for promoting psychological well-being under the pandemic. Furthermore, there is a need to promote resilience at different levels. As pointed out by Rania et al. (2022a), “empowerment is an individual and collective growing process” (p. 2). Hence, besides individual resilience, focusing on family resilience and societal resilience is also important.

Certain limitations are identified in the present study. First, single informant self-report measures were employed in the investigation, which may be biased due to social desirability and may not accurately represent family dynamics and child development (King and Bruner, 2000). Multi-informant measurements, comprising both parent and child reports, would provide a more comprehensive picture. In addition, the sample was recruited from China’s Sichuan region, which has diverse cultural and geographical conditions. The present study only took ethnicity into account. We suggest that future studies should consider other factors, such as language, socioeconomic status and cultural adaptation (e.g., Kennedy and Hue, 2011), to comprehend the complex interaction between cultural and individual factors that may affect family functioning and child development.

## 5. Conclusion

The present study purported to explore both the concurrent and longitudinal impacts of family functioning on children’s resilience, as well as the mediating impact of satisfaction with life in the family functioning-resilience relationship. In line with the research hypotheses, results indicate that family functioning concurrently and longitudinally predicts children’s resilience and support the mediation role of life satisfaction. This suggests the need for family-based interventions that promote a more supportive and stable family environment for children to grow and develop. There is also a need to nurture life satisfaction in adolescents.

## Data availability statement

The raw data supporting the conclusions of this article will be made available by the authors, without undue reservation.

## Ethics statement

The studies involving human participants were reviewed and approved by the Ethics Committee of Sichuan University (Approval code: K2020025; Approval date: 31 July 2020). Written informed consent to participate in this study was provided by the participants’ legal guardian/next of kin.

## Author contributions

DS: conceptualization and funding acquisition. DS and DD: methodology and formal analysis. DS, DD, LZ, and LT: investigation. DD: data curation. DS, DD, and LT: writing – original draft preparation and writing – review and editing. DS and LZ: supervision and project administration. All authors have read and agreed to the published version of the manuscript.

## Funding

This work was financially supported by Wofoo Foundation and the Research Matching Fund of the Research Grants Council (R-ZH4Q).

## Conflict of interest

The authors declare that the research was conducted in the absence of any commercial or financial relationships that could be construed as a potential conflict of interest.

## Publisher’s note

All claims expressed in this article are solely those of the authors and do not necessarily represent those of their affiliated organizations, or those of the publisher, the editors and the reviewers. Any product that may be evaluated in this article, or claim that may be made by its manufacturer, is not guaranteed or endorsed by the publisher.
